# Structural and functional characterization of a porcine intestinal microbial ecosystem developed in vitro

**DOI:** 10.1038/s41598-025-10144-5

**Published:** 2025-07-10

**Authors:** LinShu Liu, Jenni A. Firrman, Adrienne B. Narrowe, Karley K. Mahalak, Johanna M. S. Lemons, Massimo Marzorati, Cindy Duysburgh, Chloë Rotsaert, Tom Van de Wiele

**Affiliations:** 1 Eastern Regional Research Center, Agricultural Research Service, United States Department of Agriculture, Wyndmoor, PA 19038 USA; 2 ProDigest, Ghent, Belgium; 3 Center for Microbial Ecology and Technology (CMET), Department of Biotechnology, Ghent University, Ghent, Belgium; 4 International Associated Labs – HoMiGut, Ghent University, Ghent, Belgium

**Keywords:** Simulator of pig intestinal microbial ecosystem (SPIME), In vitro microbial community, SCFA, Bile salt conversion, Gut microbiome, Metagenomics, Microbiome, Metabolomics

## Abstract

The mammalian digestive tract harbors a vast microbial community that has the potential to modulate numerous health-related processes. Multicompartment dynamic gut models have been developed to study microbial communities in a controlled environment. To verify the assumption that the experimental results produced in vitro in a mechanical device would be highly similar to those obtained from an in vivo study, in this study fecal samples from four pigs were inoculated in a simulator of the porcine intestinal microbial ecosystem (SPIME) and cultured until reaching steady state. The composition and structure of the resultant microbial communities, and the metabolites produced were compared with those harvested from the intestine of the same pigs. Taxonomic abundance identification based on shallow shotgun metagenomic sequencing revealed only 12.1% of species or 15% of metagenome-assembled genomes (MAGs) being shared across the colon compartments of the source pigs and the SPIME. Despite these overwhelming compositional shifts, higher functional conservation was indicated as measured by functional richness, MAG-level traits, CAZymes, and untargeted metabolomics. Environmental selection and bacterial functional redundancy were considered the two key elements in microbial compositional shifts and functional preservation.

**Supplementary Information:**

The online version contains supplementary material available at 10.1038/s41598-025-10144-5.

## Introduction

Approximately 10^13–14^ bacterial cells inhabit the mammalian gastrointestinal tract (GIT) where they regulate a number of digestive and health-related processes^1–4^. Research on the gut microbiota’s contribution to health and disease and the application of this knowledge for therapeutic benefit is increasingly gaining scientific and industrial attention^4–8^. Several approaches are currently utilized to study the gut microbiota^9^. The use of fecal samples is still the most popular, as it is simple, quick, and inexpensive. The drawback is that the information it can provide is limited and does not represent the distinct composition of any of the different colon regions^10–12^. The interaction between the gut microbiota, food, and non-food components can also be evaluated by analyzing metabolites from feces, urine, blood, or other organs. This approach is growing as new analytical technology and instruments are developed, but, it only provides indirect information, and the results can be greatly influenced by the timing and method of sample preparation, such as the period from food digestion to sampling, sample collection and storage conditions, etc^13–17^. The use of animal models is another powerful way to investigate the impact of diet, medicines, and chemicals on the gut microbiota^18–21^. Pigs, in particular, are an excellent model for humans due to the similarities in physiology, genetics, and gut microbial ecology. However, the use of animal models is constrained by ethical issues, the complexity of the gut microbial ecosystem, and the multiple challenges associated with accessing in vivo sampling sites. These factors combined prompted the development of in vitro approaches for gut microbiome and metabolome research^4,21^.

Several in vitro models have been developed for continuous in vitro gut microbial culture, using either fecal sample or synthetic microbial cocktail as inoculum^22–26^. The single-vessel models currently available for use are too simple to reflect the physiological environment of the GIT that differentiated to different regions and phases with distinct characters, and thus the use of single-vessel model is unable to recapitulate the complexity and peculiarity of the gut microbial communities inhabit longitudinally through the GIT^27,28^. An ideal in vitro model is a multi-compartmental unit that recapitulates the different GIT regions, harbors communities that develop from both the luminal and mucosal phases and replicates the physiologic conditions of the GIT^20,29^. These simulators can be inoculated with fecal samples which, when cultured under physiological conditions, mature into an in vitro gut microbial community^23–27^. This community can be used to evaluate the impact of various perturbations on the composition and metabolism of the microbiome. A benefit of these models is that sampling can be performed at any time from any compartment (representing different colon regions), enabling rapid detection of changes. In addition, by excluding the mammalian host components from the in vitro system, the results can be confidently and solely attributed to the response of the gut microbial community; on the other hand, the lack of the eukaryotic component and the disconnection from the main circulatory system alter microbial growth and metabolism^30^.

Nevertheless, there remains a lack of information detailing the similarities and differences between the community composition developed in vitro and the community of origin found in vivo^31,32^. The present study describes an effort to make a Simulator of Porcine Intestinal Microbial Ecosystem (SPIME) to better understand the relationship between the gut microbial ecosystem derived from porcine feces within the simulator and the in vivo counterparts collected from the pig GIT. The same four pig donors were used for both the in vitro and in vivo analysis. The research focused on the difference in composition and functionality of the microbial ecosystem that developed within the SPIME compared to the in vivo situation, as well as the relationship between the compositional and functional changes.

## Materials and methods

### Animals and materials

The study was conducted in accordance with the ethical standards and recommendations for accommodation and care of laboratory animals covered by the European Directive 2010/63/EU on the protection of animals used for scientific purposes and the Belgian royal decree KB29.05.13 on the use of animals for experimental studies and reported in accordance with ARRIVE guidelines as relevant. Use of fecal microbiota for in vitro experiments was approved by Ghent University’s ethical committee and was registered as B6702018-36318.

Four nine-week-old, TopigsNorsvin x German Piétrain piglets (ILVO, Melle, Belgium) were used in this experiment. The piglets were acclimated for 3 weeks and housed as described in Rotsaert et al.^33^ On day 21, feces from each pig were collected in a sampling box together with an Anaerogen bag to remove all oxygen from the environment and cryopreserved following the same procedure as described in Rotsaert et al.^33^ Immediately after fecal collection, the animals were weighed and euthanized by electronarcosis, followed by exsanguination and samples from both the luminal phase and mucosal phase of the proximal and distal colon regions were collected and stored for metagenomic sequencing, analysis of short chain fatty acids (SCFA), bile salt profiling, and untargeted metabolomics. These samples represent the in vivo porcine microbiome which is compared to the in vitro microbiome derived from the fecal samples.

The medium used for the in vitro experiment was standard pig feed that was also used to feed the animals during 3 weeks from housing to termination (Supplementary Table 1, adapted from Rotsaert et al.^33^. ) The predigested pig feed, chyme, harvested at the end of small intestine was prepared as described in a previous publication^34^ and ProDigest’s operation manual. Briefly, feed was treated with pepsin at pH 2 for 2 h under vigorous stirring at 39 °C, then treated with a group of bioactives consisting of bile salts and small intestinal enzymes at pH 6.8–7.2 for 1.5 h at 39 °C. The slurry thus produced was dialyzed against Na_2_CO_3_/NaHCO_3_ solution, reconstituted to pH 7.0 for using as feed for the SPIME^33^.

### Experimental design and operation

**Fig. 1:**
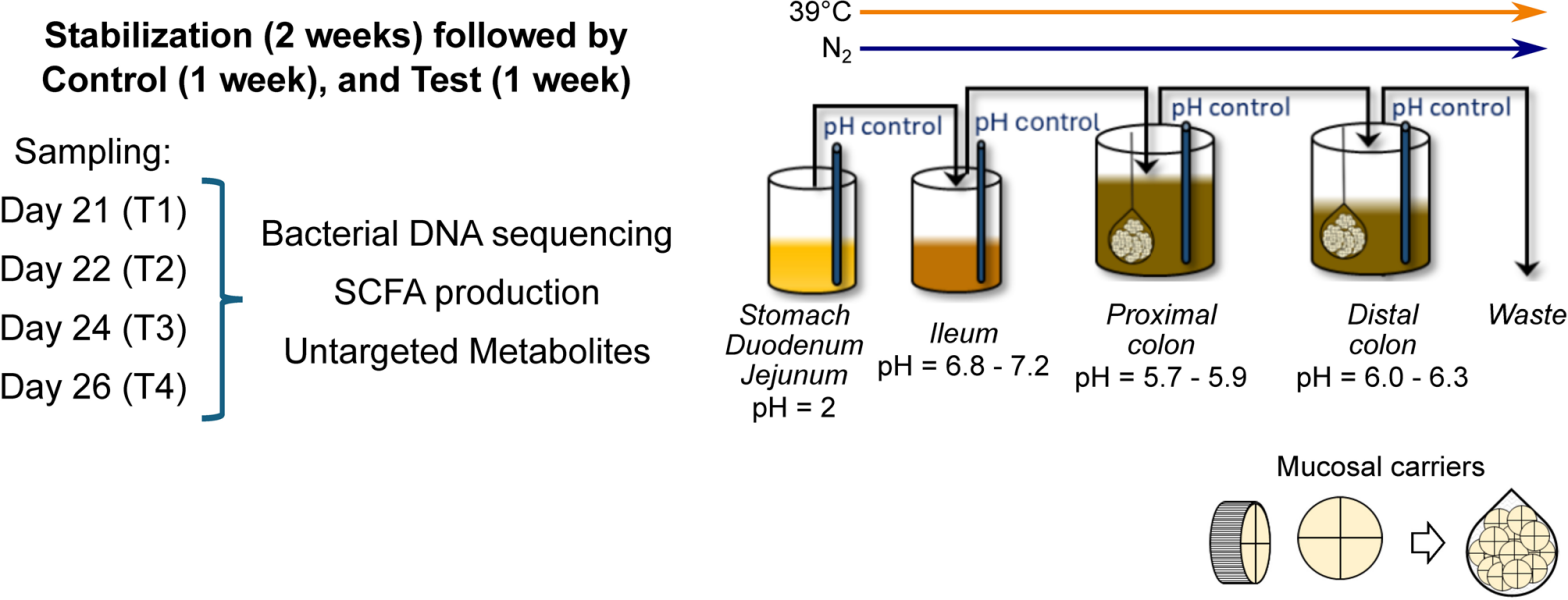
Experimental design. Each unit contains 3 bioreactors for predigested feed storage, proximal colon, and distal colon, separately; each colon compartments has a luminal phase and a mucosal phase; the system was maintained anaerobic at 39 °C.

The design and operation of the present experiment is illustrated in Fig. 1. A SPIME was used for the in vitro experiment, indicating that the set up includes a mucosal phase in addition to the standard luminal phase. Four SPIME units were arranged side by side, each pig assigned to one unit. Each unit consisted of four compartments, two representing the upper GI tract used to mimic the passage of predigested pig feed, and the other two compartments representing a combined caecum and proximal colon (PC) and a distal colon (DC). The latter two bioreactors were inoculated with pig-derived fecal microbiota which were allowed to adapt to the prevailing conditions in the cecum/proximal colon and distal colon compartments.

The mucosal environment, allowing simulation of the colonization of the mucus layer, was incorporated in the bio-reactors, as described in Van den Abbeele et al.^35^ In brief, 5% porcine mucin type II (Sigma-Aldrich) and 1% agar (VWR Belgium) were dissolved in distilled H_2_O, boiled and pH was adjusted with 10 M NaOH to 6.8. Subsequently, microcosms were coated with this mucin-agar and 28 microcosms were added per colonic reactor. Half of the microcosms were replaced regularly by fresh sterile ones to simulate renewal of the mucus layer. Mucin was collected by centrifugation for further analysis.

The chyme was degassed and slowly transferred to the colon compartments. These colon compartments are continuously stirred with constant volume and pH control. The PC compartment works with a delayed fill-and-draw principle i.e., when the chyme is transferred to this compartment, there is a static incubation period of 3 h, mimicking the caecum, followed by a slow transfer from the cecum/proximal colon to the distal colon compartment. The two colon compartments were maintained at pH 5.7–5.9 for PC and pH 6.0–6.3 for DC and consistent at 39 °C, the system was kept anaerobic by flushing with N_2_. On day 21 after inoculation, samples were taken from bioreactors every other day for 4 consecutive time points (T1, T2, T3, and T4) for analysis of bacterial composition and metabolites.

### DNA extraction, library preparation, and sequencing

Shallow shotgun sequencing was performed by CosmosID (Germantown, MD) according to a standard protocol as previously described^36–38^. Briefly, DNA was isolated using the QIAGEN DNeasy PowerSoil Pro Kit, according to the manufacturer’s protocol (QIAGEN, Germantown, MD, USA.) Extracted DNA samples were quantified using Qubit 4 fluorometer and Qubit™ dsDNA HS Assay Kit (Thermofisher Scientific, Waltham, MA, USA.) DNA sequencing libraries were prepared using the Nextera XT DNA Library Preparation Kit (Illumina, San Diego, CA, USA) and IDT Unique Dual Indexes with total DNA input of 1ng. Genomic DNA was fragmented using a proportional amount of Illumina Nextera XT fragmentation enzyme. Unique dual indexes were added to each sample followed by 12 cycles of PCR to construct libraries. DNA libraries were purified using AMpure magnetic Beads (Beckman Coulter, Indianapolis, IN, USA) and eluted in QIAGEN EB buffer. DNA libraries were quantified using Qubit 4 fluorometer and Qubit™ dsDNA HS Assay Kit. Libraries were then sequenced on an Illumina HiSeq X platform 2 × 150 bp to a target depth of ~ 3 M read pairs per sample.

### Taxonomic and functional profiling

Raw shotgun metagenomic sequencing reads were preprocessed for adapter removal and quality trimming and filtering using BBDuk v38.7 (https://sourceforge.net/projects/bbmap/) with the parameters (k = 31, hdist = 1, ftm = 5, qtrim = r, trimq = 10.) Trimmed reads were subsequently filtered to remove host DNA- derived reads using BBDuk with the Sus scrofa reference genome Sscrofa 11.1 (GCF_000003025.6_Sscrofa11.) Trimmed, filtered reads were used as input to perform read-based taxonomic assignment and relative abundance estimation using MetaPhlAn v 4.0.6 using the mpa_vOct22_CHOCOPhlAnSGB_202212 reference database^39^. Taxonomic and abundance profiles for all samples were merged and used as a taxa/samples table for downstream analysis. Read-based functional profiling was performed using HUMAnN v 3.6^40^, generating KEGG ortholog classifications and estimated KO abundances^41–43^.

### Alpha- and beta-diversity analysis

Alpha and beta diversity were calculated using the MetaPhlAn utility script calculate_diversity.R. For alpha diversity, taxonomic richness and Shannon Diversity index were calculated using both the read-based taxonomic and functional (KO) profiles. Testing for significant differences among treatment and timepoints was performed using thepairwise Wilcoxon text with Benjamini Hochberg multiple testing correction using the rstatix R package^44^. Beta diversity was calculated as the weighted and unweighted UniFrac distance. PERMANOVA testing for significant clustering was performed using the pairwiseAdonis2 R package with subsequent Benjamini Hochberg* p*-value correction within each figure panel^45^.

### Metagenome assembly and annotation

Trimmed filtered reads were co-assembled by pig using MEGAHIT v1.2.9, with all sequencing reads derived from the in vivo and in vitro samples from each pig used as input, to produce four co-assemblies^46^. Following assembly, reads were mapped back to the contigs from each assembly using BBMap v38.7 (https://sourceforge.net/projects/bbmap/), and binned using MetaBAT2^47^. Subsequent assemblies were dereplicated using dRep v 3.2.2^48^. The final set of dereplicated metagenome-assembled genomes (MAGs) were annotated using DRAM, microTrait and dbCAN3 (run_dbcan v4.1.1)^49–51^.

### SCFA quantification

SCFA were extracted as previously described^52^. Briefly, SCFAs were extracted using diethylether and detected on a GC-2014 capillary gas chromatograph (Shimadzu, Hertogenbosch, Netherlands) equipped with a GC SGE capillary column (30 mm × 0.32 mm ID-BP 21 × 0.25 μm, Achrom, Machelen, Belgium), a flame ionization detector and split injector.

### Untargeted metabolomics

Untargeted metabolomics was performed on luminal fluid samples from both the proximal and distal colons of the four selected pigs and on the corresponding in vitro colon region samples (PC and DC). Metabolomics was performed by Metabolon (Durham, NC, USA) using the following protocol: Samples were extracted and separated as hydrophilic and hydrophobic phases. Each extract was analyzed a minimum of 4 times, using a different and independent platform each time to gain the maximum amount of data; two separate reverse phases ultra-high performance liquid chromatography (RP/UPLC-MS/MS) associated with tandem mass spectroscopy with either positive or negative ion mode electrospray ionization, as well as hydrophilic-interaction chromatography (HILIC/UPLC-MS/MS) The scan range used for these analyses covered at least 70–1000 m/z, the mass accuracy matching threshold < 10 ppm. Metabolites were annotated using the proprietary Metabolon database and median-scaled per metabolite. Missing values were imputed as the minimum median-scaled value for each metabolite and log transformed. Batch correction was not required due to the small number of samples which were run in a single instrument batch.

### Statistical analysis and visualization

Statistical analyses and visualizations were conducted using R/RStudio (v.4.1.3)^53^ using the packages: tidyverse (v.1.3.1)^54^, vegan (v.2.6-2)^55^, ape (v.5.6-2)^56^, ggvenn^57^, pheatmap^58^, and rstatix^44^. Testing for significant differences among the ratios of SCFAs within each color region was performed using paired t-tests on the proportion of each SCFA within each sample with Benjamini Hochberg multiple testing correction. Figures were refined using Inkscape v1.1 (inkscape.org.)

## Results

### Significant differences in microbial composition between the in vivo and SPIME communities

**Fig. 2:**
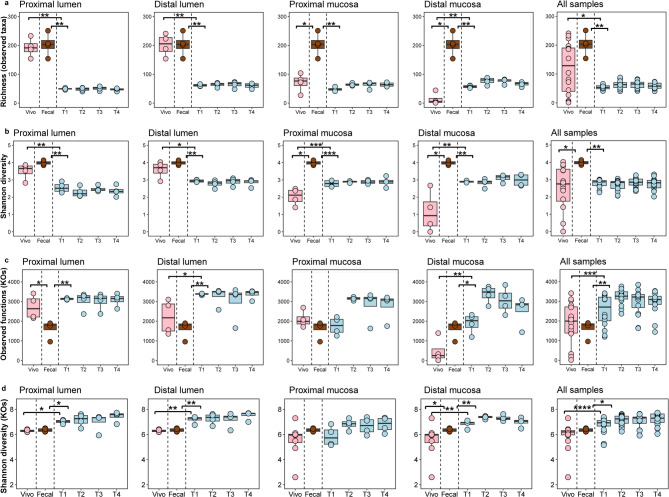
Alpha diversity measures differ by sample source. (**a**) Species richness based on number of taxa observed. Boxplots represent the values for the four pigs. The x-axis indicates the in vivo sample, fecal sample, and the mSHIME at 4 timepoints. Panels represent the proximal and distal luminal and mucosal samples, and all samples combined; (**b**) Shannon’s diversity index: Panel arrangement and x-axis as in panel a.; (**c-d**) Functional richness based on estimated KEGG ortholog abundances (KO), and Shannon’s diversity index (KO) Panel arrangement and x-axis as in panel a. Statistical significance across experiments/sample types was tested using the Wilcoxon test with Benjamini Hochberg false discovery rate correction. Adjusted *p*-value * <0.05, ** < 0.01, *** < 0.001.

Shallow shotgun sequencing was used to detail the microbial composition and structure of the in vivo samples obtained from the four pigs’ colons, feces, and the corresponding communities that developed in the SPIME. Using the resulting taxonomic profile, alpha- and beta-diversity were calculated. The first alpha-diversity metric measured was taxonomic richness (TR_α_). As shown in Fig. 2A, there was a statistically significant difference in TR_α_ observed between the in vivo and in vitro communities (W = 327.5, q = 0.04) as well as between the in vitro communities and the fecal samples (W = 356, q = 0.003.) Upon cultivation, TR_α_ dropped significantly for both the luminal and mucosal communities in both the proximal (PC) and distal colon (DC) regions, followed by non-significant variations over time, and between the PC and DC of both the luminal and mucosal phases. In vivo samples differed significantly from fecal samples in the mucosal, but not luminal phase of both colon regions.

Shannon’s Diversity Index showed significant differences when comparing the in vivo to the in vitro communities in the individual locations, but it did not differ significantly between the in vivo and in vitro communities across all samples (Fig. 2B). Furthermore, for the in vitro communities, the Shannon Index values did not differ significantly across the four consecutive time points (T1 to T4),. Another alpha-diversity metric, functional richness (FR_α_) was calculated, which as used here represented the sum of estimated KEGG Ortholog (KO) abundances in the samples regardless of taxon of origin. The differences in FR_α_ values of both the in vivo and in vitro communities were visible in the PC (both luminal and mucosal), and significantly higher in vitro than in vivo in DC (lumen adjusted * p*-value *p* < 0.05, mucosa adjusted * p*-value < 0.01) (Fig. 2C) despite a drop for the feces. Since the in vivo communities had higher taxonomic richness than the in vitro communities (Fig. 2A), we have.

$${({\text{F}}{{\text{R}}_a}/{\text{T}}{{\text{R}}_a})_{{\text{pig}}}}<{({\text{F}}{{\text{R}}_a}/{\text{T}}{{\text{R}}_a})_{{\text{culture}}}}$$

that indicates that more taxa share same KO pathway in the SPIME community than the in vivo samples. The Shannon diversity of KO, as shown in Fig. 2D, is statistically higher in vitro than in vivo samples (adjusted * p*-value < 0.00001), except for the proximal mucosal samples, meaning that the SPIME community possesses a greater functional diversity than those in piglet’s GIT.

**Fig. 3:**
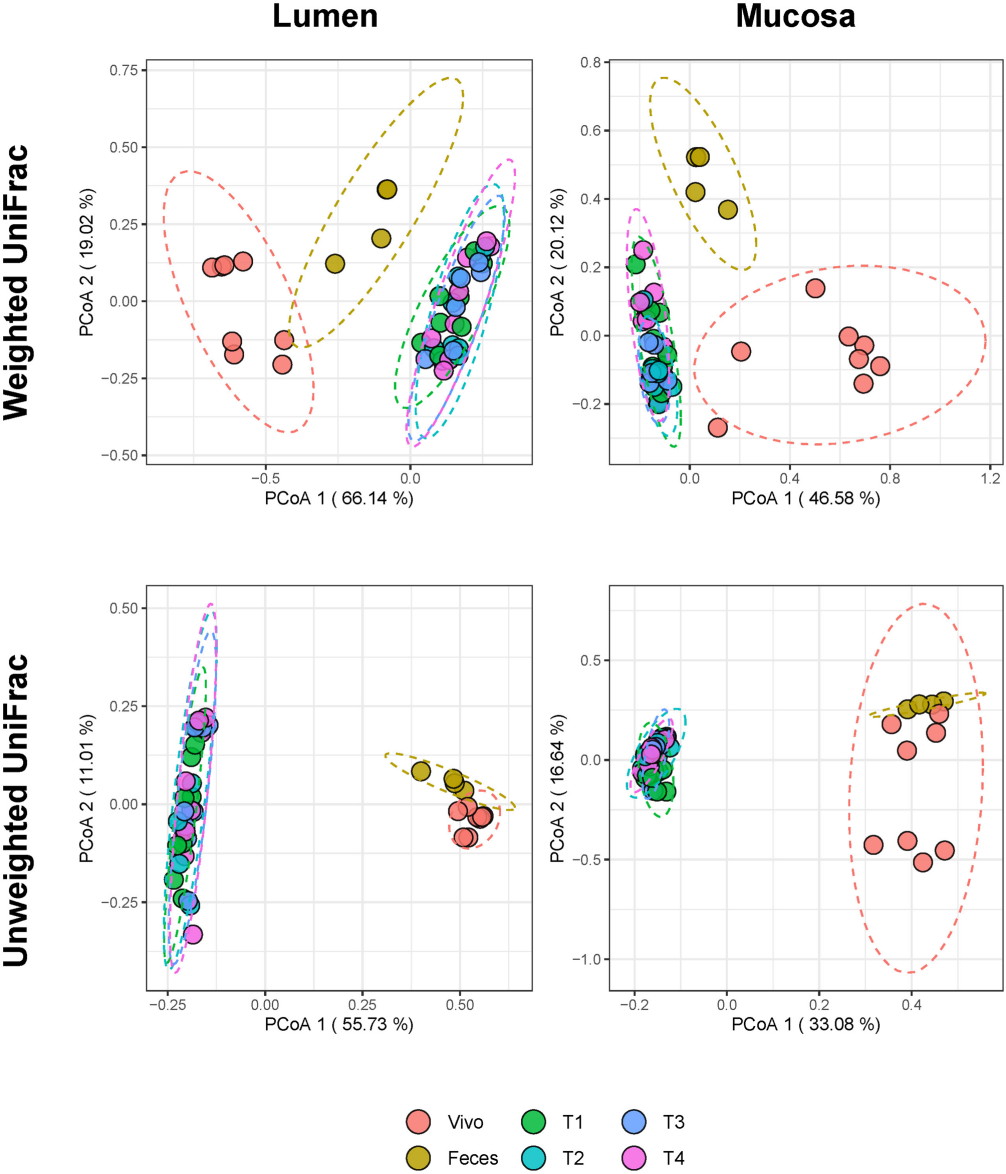
Samples cluster by sample source. Principal Coordinate Analysis (PCoA) plot of weighted UniFrac (top) or unweighted UniFrac (bottom) distances. Lumen and Mucosa samples are plotted separately with the same fecal samples included in each plot. In vivo, in vitro, and fecal samples all cluster together with their respective type (PERMANOVA *p* < 0.05) but the in vitro samples do not differ by timepoint.

The beta diversity of the gut microbiota in in vivo, feces, and SPIME communities were evaluated using weighted and unweighted UniFrac distances, and visualized using Principal Coordinate Analysis (PCoA), as shown in Fig. 3. For both the luminal and mucosal communities, the ordination of weighted UniFrac distances demonstrated a statistically significant divergence between in vivo and in vitro conditions. The fecal samples were positioned intermediately between the two clusters, yet also differed significantly from them (Supplementary Table 2). There were no significant differences among the SPIME communities and/or among the four time-points analyzed for either the luminal or mucosal communities. Overall, the in vitro communities clustered tightly together. This profile was also observed for the unweighted UniFrac distance plots, yet here the distance between the feces and the in vivo communities was less pronounced, though still significantly distinct in the luminal samples.

**Fig. 4:**
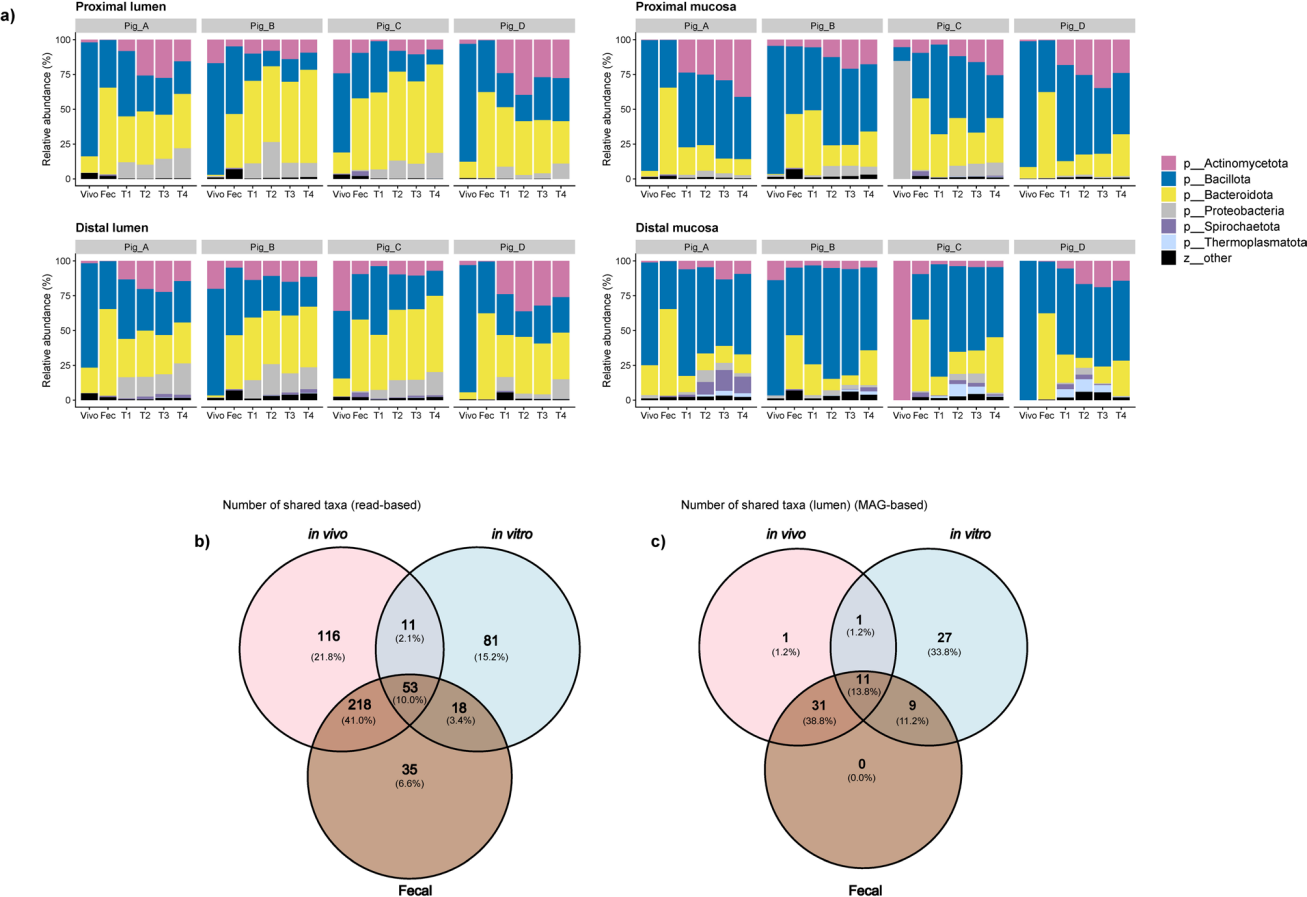
Phylum level abundances display differences by sample site and type with low overlap at the species level. (**a**) Bar plots of phylum-level relative abundances, the 6 most abundant phyla are listed individually with the remaining phyla combined as ‘other’; (**b**) Venn diagram showing the overlap of taxa at the species level (read-based classification; (**c**) Venn diagram showing overlap of MAGs across the three sample types.

Analysis of both alpha and beta diversity metrics revealed significant divergence in the gut microbial communities between the in vivo conditions and the SPIME, indicating a sharp change in the composition and ratio of the taxa between the in vivo and the in vitro communities. Next, we delved to the change of taxonomic distribution from phylum down to species level to identify the drivers of this divergence (Fig. 4A). Of the 21 phyla identified, most taxa belonged to only four, Bacillota, Bacteroidota, Actinomycetota, and Proteobacteria which combined accounted for a relative abundance of 96% in the feces, 95% in the mucosal communities, and 98% in the luminal communities. Beyond the bacteria, there were six archaeal species, among them *Methanomethylophilus alvus* was present at > 1% in the mucosal environment of the distal colon region, and other five had a maximum relative abundance < 1% in any community.

A dynamically stable gut microbial community was established in SPIME in the present research as evidenced by minimal variation in bacterial composition among the four consecutive time-point measurements (Fig. 4A), that is consistent with previous reports^31,59,60^. Fig. 4A also shows no apparent compositional change between the proximal and the distal regions for the in vivo communities; similarly, no substantial compositional change occurred between the PC and DC for the in vitro communities, but obvious and expected differences persisted between the luminal and mucosal communities; these results were consistent with the alpha- and beta- diversity analysis results (Figs. 2A,B and 3).

Next, we compared the bacterial compositions between the in vivo community, the feces, and the SPIME community. In vivo, the phylum Bacillota (43%) was on average the most abundant followed by Bacteroidota (34%). However, the fraction of Bacteroidota increased in both the feces and SPIME communities as Bacillota decreased while Actinomycetota were more prevalent in all SPIME communities compared to the in vivo communities and feces (Fig. 4A). At the lower taxonomic levels, there were larger differences in composition between the in vivo and in vitro communities (Supplementary Fig. 1). At the family level, members of the phyla Bacillota: *Lactobacillaceae*, *Streptococcacea*e, *Oscillospiraceae*, *Lachnospiraceae*, and *Ruminococcaceae* presented primarily in the in vivo communities, and to a lesser extent in the feces and in vitro communities. However, the family *Bacteroidaceae* of Bacteroidota increased in vitro relative to in vivo. At the genus level, all genera of Lactobacillaceae totally disappeared, genus *Mitsuokella* of family *Selenomonadaceae*, *Phascolarctobacterium_A* of *Acidaminococcaceae*, *Bulledia of Erysipelotrichaceae*, and *Evtepia* of *Oscillospiraceae*,, increased visibly, but not significantly in the distal reactors by the last timepoint compared to the in vivo samples (MaAsLin3 * p*-values < 0.005, q-values = 0.08), while in the proximal reactors, the genus *Megasphaera* was significantly increased in vitro (MaAsLin3 * p*-value 0.0002, q-value 0.004.) The genus *Prevotella*, from the *Prevotellaceae* family of phylum Bacteroidota represented by 37 species (Supplementary Fig. 3), was among the most abundant taxa identified in the SPIME. The genus *Prevotella* increased significantly in the distal reactors (*p* = 0.0002, q = 0.045) and visibly but not significantly in the proximal reactors (*p* = 0.0007, q = 0.069.) At the species level, read-based taxonomic and abundance classification identified 50.1% of bacterial species were shared by the in vivo and the fecal samples, and only 12.1% or 64 species were shared between the in vivo and in vitro communities (Fig. 4B, Supplementary Table 3). Of these, only 53 species (10%) were also detected in the in vivo samples, feces, and SPIME community.

To obtain more information from a genome-centric perspective, MAGs were assembled. The group of MAGs shared across the in vivo communities, feces, and in vitro communities were identified and are shown in Fig. 4C. Of 80 MAGs found in luminal communities, 11 were shared between the in vivo and in vitro communities and the feces (13.8%), and 12 were shared by the in vivo and SPIME (15%), while 79.2% (42 in 53) were shared between the in vivo samples and the fecal samples. These data highlight the critical “bridge” role of the fecal samples as the inocula in establishing an in vitro gut microbial ecosystem in SPIME. In the following sections, the impact of the structural divergence on metabolism and functionality was considered, specifically the capability of the bacterial community developed in the SPIME to perform the same functions as the native gut microbial community.

### SPIME microbial communities retain in vivo metabolic profiles

**Fig. 5:**
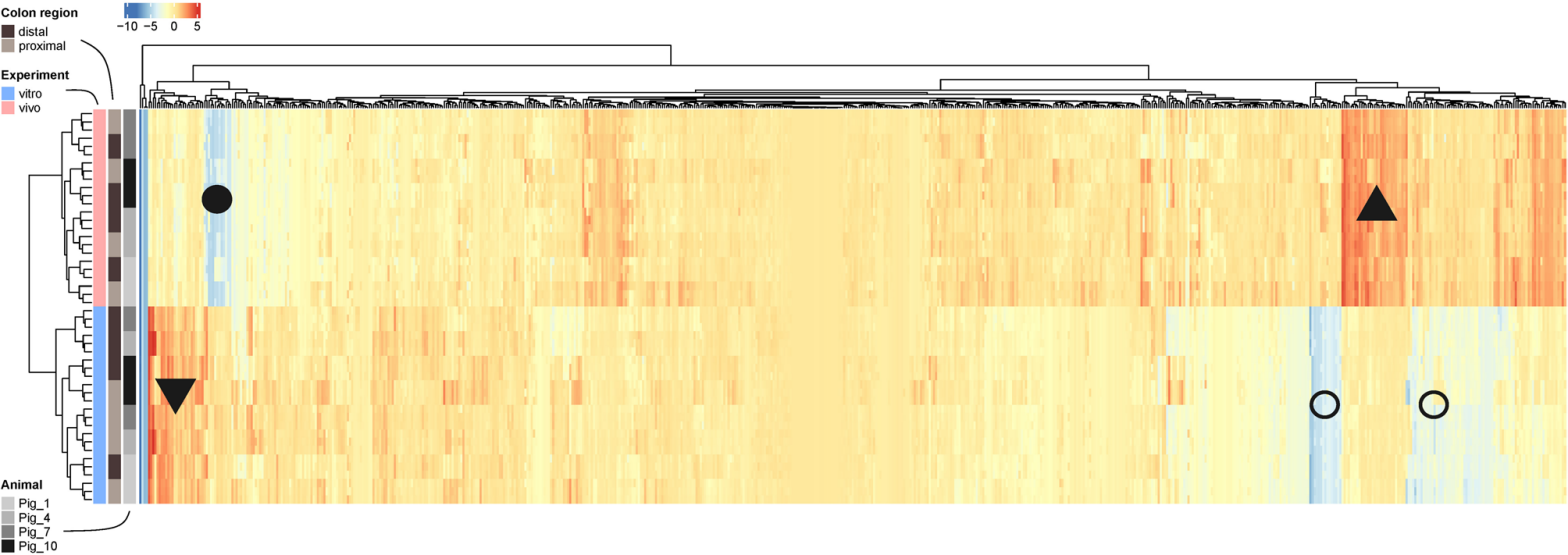
Metabolite abundances reflect sample of origin and suggest quantitative differences only. Heatmap of metabolite abundances. Rows represent samples and columns represent metabolites (individual labels not shown.) Triangles and circles represent areas discussed in the text. All heatmaps are hierarchically clustered (two-way). Normalized peak area abundances of selected bile salts from untargeted metabolomics data.

Figure 5 and Supplementary Table 4 show the 667 annotated metabolites identified across the different regions of the porcine colon and within the SPIME. The heatmap shows that the differences in metabolites between the in vivo and in vitro communities were quantitative, and not a difference in their presence or absence. Overall, 95.6% ofmetabolites identified from the in vivo communities were also identified in at least one of the SPIME communities with a few exceptions that are indicated in the figure. For example, in the area marked with ▲, phenylalanine, indole-carboxylate, and other tryptophan metabolism products were enriched in vivo; in the area marked with ▼, more glycocholate and ursodeoxycholate were detected in the SPIME communities than the in vivo communities. In addition, there were metabolites detected at very low abundance, such as ribothymidine and cytidine in vivo (●), or glycerol-phosphate and orotate in vitro (○).

**Fig. 6:**
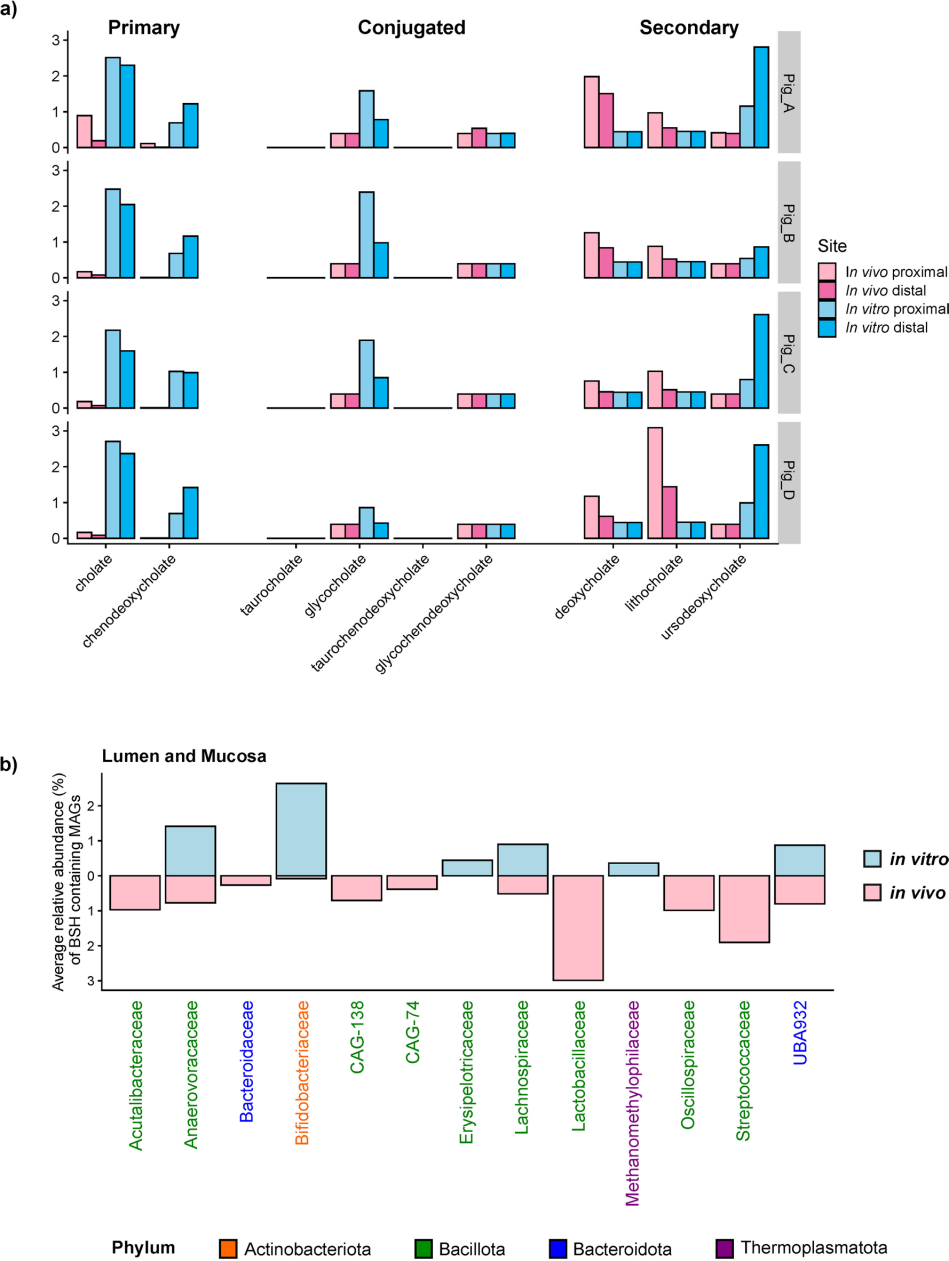
Potential for bile salt conversion is largely retained in the in vitro system. (**a**) Distribution of bile salts from untargeted metabolomics; (**b**) Bar graph showing abundances of taxa containing bile salt hydrolase genes, grouped at the family level.

The amounts of individual bile salt measured were summarized in Fig. 6A. The difference in the cholate and chenodeoxycholate content between the in vivo and in vitro communities was significant (adjusted * p*-values < 0.0001). Taurocholate and taurochenodeoxycholate were nearly completely undetected in both the two communities. It was also found that the secondary bile acids, deoxycholate, lithocholate, and ursodeoxycholate were more variable in their abundance pattern for the two communities, though the latter two did differ significantly between in vivo and in vitro conditions (adjusted * p*-values *p* < 0.0001 and *p* < 0.001 respectively.)

**Fig. 7:**
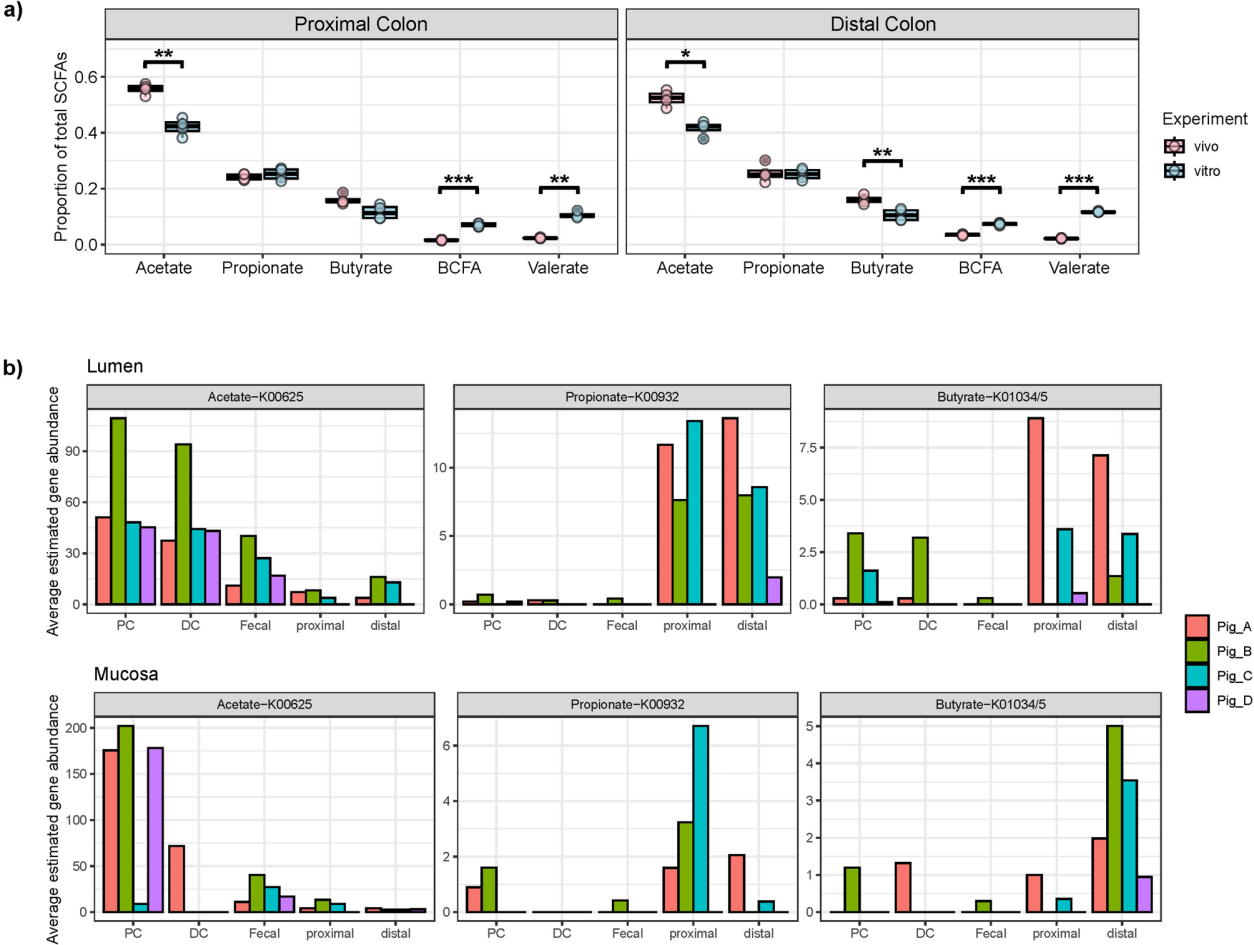
Potential for short chain fatty acid production is largely retained in the in vitro system. (**a**) Proportional abundance of five SCFA types relative to total SCFAs, comparing proportions between in vivo and in vitro samples in both the proximal and distal colon regions. Stars indicate significant paired t-tests of difference in proportions across experiments; (**b**) Estimated abundances of genes (KO) associated with SCFA production K00625 for acetate, K00932 for propionate, and K01034/K010355 for butyrate.

In the present study, bacteria containing bile salt deconjugation genes were detected across 13 families with different abundances, among them two were only detected in the SPIME, seven were only found in vivo, and four were common between them (Fig. 6B). These 13 families belong to three bacterial phyla and one archaeon. Despite the metabolite profiles suggesting bile salt conversions were occurring, the *bai* genes responsible for secondary bile salt formation were not detected. Figure 7 compares the proportional abundance of each SCFA between the in vivo and the SPIME models in the PC and DC regions. The SCFA production displayed the same pattern for both systems: acetate > propionate > butyrate > other SCFAs. However, there were significant differences at the levels of SCFAs between the in vivo and in vitro communities detected, i.e., the proportion of acetate in vivo was significantly higher than in vitro (paired T-test adjusted * p*-value 0.01 in DC and 0.009 in PC), and the proportion of valerate and BCFA were higher in vitro (valerate: paired T-test adjusted * p*-value 0.0002 in DC and 0.002 in PC; BCFA: adjusted * p*-value 0.0004 in DC and 0.0006 in PC). The production and distribution of propionate and butyrate varied by pig, but only butyrate was statistically different between the in vivo and in vitro communities and only in the DC region (paired T-test adjusted * p*-value 0.003 in DC.) Correlatively, genes capable of acetic acid production (K00625) were more prevalent in the in vivo communities, while higher gene abundances, K00932 and K01034/5, were observed in the in vitro communities for propionate and butyrate, respectively (Fig. 7B).

Figure 5 provides a snapshot of the metabolic activity of the SPIME microbial community based on direct measurements. Next, we evaluated the potential capabilities by genome-centric prediction. Previously defined microbial traits were used to describe the interaction of an organism with the environment and are inferred from genes and pathways^61^. Supplementary Fig. 4 plots the relationship of traits (in rows) against individual communities (in columns). Overall, most of the traits identified in vivo persisted in the SPIME. There are two exceptions, one is the lack of specific stress tolerance function found in vitro (red arrow), due solely to the loss of three lactic acid bacteria (Supplementary Fig. 1), *Limosilactobacillus reuteri*,* Ligilactobacillus salivarius*, and *Streptococcus hyointestinalis*. Of note, other pathways possessed by these species, such as carbohydrate depolymerization, were retained in the community (blue arrow) and this persistence could be attributed to the contribution of these traits by other MAGs. This example demonstrated that the loss of the three taxa had limited impact on functional potential. The other exception was a gain in anaerobic respiratory capacity in vitro (yellow arrow) which could be attributed to a single *Pseudomonas aeruginosa* MAG.

The abundance of carbohydrate-active enzyme (CAZyme) gene families as well as the community carbohydrate substrate range was estimated based on MAGs. As shown in Supplementary Fig. 5, the highest enzymatic potential was detected for starch, sucrose, glucans, and other glycans both in vitro and in vivo, followed by polyphenol and some sugars containing alpha-rhamnoside linkages. In contrast, lower enzymatic potential was predicted for substrates such as glucomannan, chitosan, galactomannan, and lignan, most of which are indigestible fibers or fiber components. Although quantitative differences were detected between the in vitro and in vivo communities for some substrates, such as xyloglucan, exopolysaccharides, and beta-glucuronan, or between individual pigs, and select SPIME communities, the results indicated that similar CAZyme profiles were present both in vivo and in vitro, even though their microbial compositions were largely different^62^.

### A fraction of the gut microbiota represents functional capability and compositional diversity

**Fig. 8:**
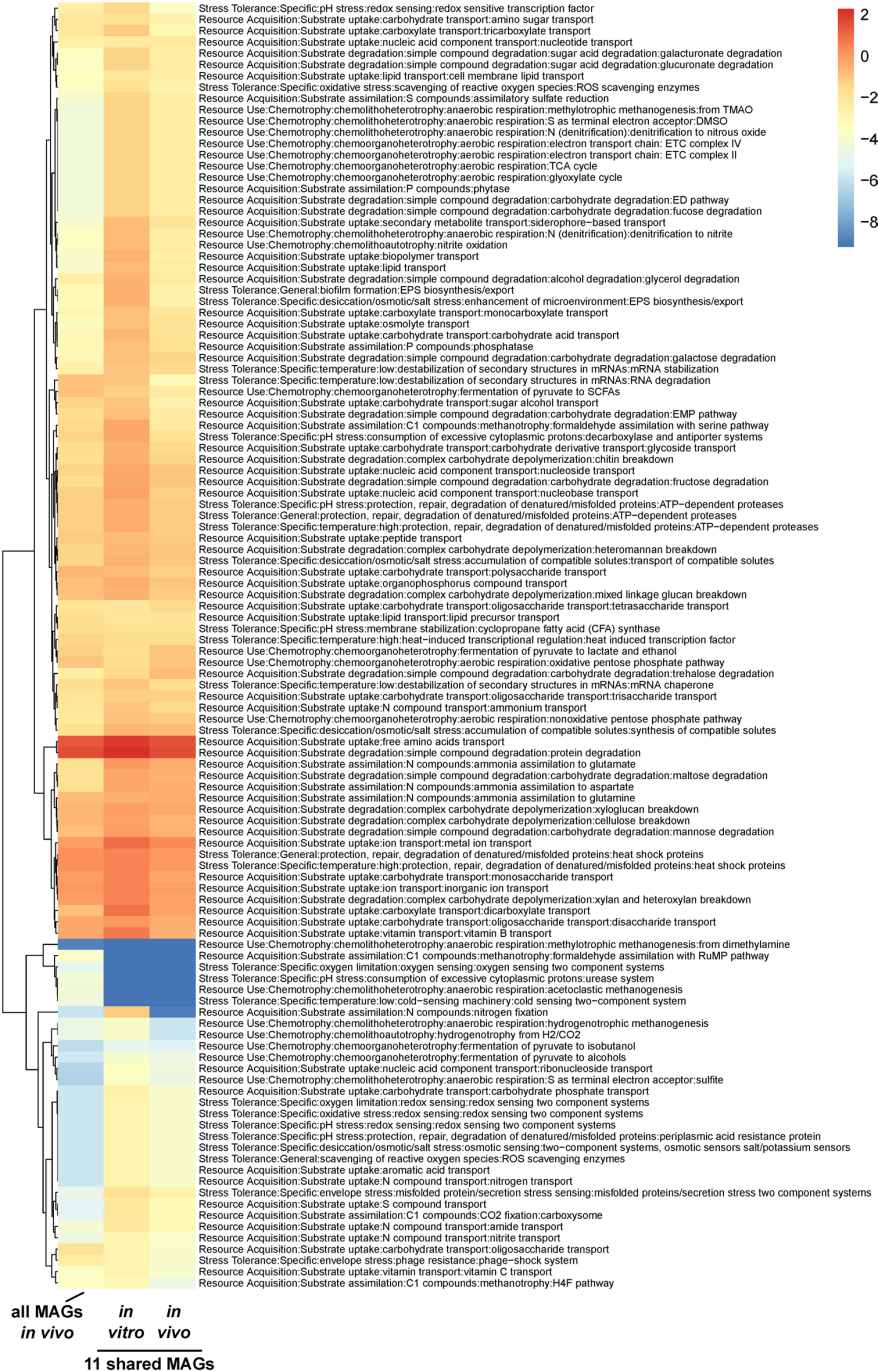
Trait-based analysis shows overall functional potential is largely captured by shared group of 11 MAGs. Heatmap showing trait abundances for the 11 MAGs shared across sample types relative to all MAGs. Columns represent sums of within-MAG trait abundance x MAG abundance in samples of that type.

Figure 8 shows a trait plot, where the left column represents the in vivo community with a full spectrum of all MAGs, the middle column portrays the trait abundance of only the 11 shared MAGs in the in vitro samples, and the right column shows the trait abundance of the 11 shared MAGs in the in vivo samples. For most areas, the columns only differ by degree and not by the presence or absence of a trait. Blue areas indicate either a lower abundance of the MAG with that trait, or a lower number of MAGs with that trait, or a combination of both. The results of the trait analysis revealed that the 11 MAGs found in the two communities possessed the same traits as the full complement of MAGs identified in the in vivo communities.

As some of these traits represent metabolic conversions that may impact the host gut ecosystem, this suggested that the overall functionality of the gut microbiome is represented by these 11 taxa, however, these trait profiles are coarse-grained, and some important functions may not be represented in this analysis. Because metagenomic assembly may not return MAGs of all the taxa present in a community, this comparison was also made using the shared species profile generated by the read-based analysis. Because trait characterization requires genomes or MAGs, the species representative from the Genome Taxonomy Database (GTDB) for each of the 64 shared species was used and the results are shown in Supplementary Fig. 6, where the top row represents the inferred abundance of the trait in the in vitro communities based on the shared 64 species, and the bottom row is for the in vivo communities. As with the MAG analysis, the 2 rows only differ quantitatively, not in the presence or absence of a trait.

The 64 shared species (based on the read-based analysis), span seven phyla (five bacterial and two archaeal) in both the SPIME and the in vivo communities; the relative abundances (RA%) of the shared species, summed by phylum, was calculated and shown in Supplementary Table 5. The RA% of the shared bacterial species was low in the in vivo communities, and lower in fecal samples, where the environmental pH, temperature and aerobic/anaerobic conditions were very different from the gut. In the in vitro samples the RA% increased as the bacterial community developed in the SPIME, which possesses conditions that are physiologically similar to the porcine GIT. Interestingly, for the in vivo communities, Bacillota and Bacteroidota were the dominant bacteria in both the species richness and RA% (Fig. 4A, Supplementary Table 3); for the in vitro SPIME communities, both Bacillota and Bacteroidota still dominated in terms of species richness. Furthermore, the species richness and RA% of Bacillota were always higher than that of Bacteroidota.

## Discussion

The inoculation of porcine fecal samples into the SPIME led to the development of unique, stable, subcommunities within the PC and DC regions of the in vitro model and within the luminal and mucosal niches of these regions that were phylogenetically similar to the starting fecal materials (Fig. 4A). Considering that no significant differences of TR_α_ and Shannon diversity index were detected between the fecal samples and the in vivo communities, that the PCoA visual distances of both weighted and unweighted UniFrac distances from the in vivo community to the fecal samples were shorter than from SPIME community (Fig. 3), the fecal materials were more similar to their donors. This was further supported by both read-based taxonomic distribution analysis and from genome-centric perspective as shown in Fig. 4B, where 53 species were found in fecal samples in the total 64 species shared by the in vivo, fecal and SPIME communities, or 11 MAG was detected in the total of 12 shared by the three; in other words, the majority species or MAG shared by the in vivo and SPIME communities were present in the fecal samples. This data indicates the bridge-like function of fecal samples between the in vivo and the in vitro communities. The composition of all subcommunities remained consistent after reaching a steady state, being consistent with previous research on developing in vitro gut microbial ecosystems within multi-compartmental simulators using fecal samples^23–27,44,45^. These results demonstrate the feasibility of developing an in vitro porcine microbial community derived from fecal samples in a multicompartment-simulator for use in gut research.

Furthermore, both alpha- and beta-diversity analyses (Figs. 2A,B) and the results of taxonomic distribution (Fig. 4A) revealed significant differences in microbial compositions between the in vivo and the SPIME communities. First, a large fraction of bacteria was lost during the transition from the in vivo community to fecal samples and then to the conditions within the SPIME; only 12.1% or 64 species, 15% or 12 MAGs were shared by the two communities (Fig. 4B). Second, the average abundance of Bacillota in the SPIME community decreased compared to the in vivo community, while the opposite trend was observed for Bacteroidota. The decreased abundance of Bacillota in vitro could be largely attributed to a loss of the lactic acid bacteria *L. reuteri*,* L. salivarius*, and *S. hyointestinalis*, while the 37 species within *Prevotella* were the most abundant taxa in the SPIME communities (Supplementary Fig. 2).

It has been reported that the change in environmental pH can impact SCFA production^63^and the loss of lactic acid bacteria may create a more favorable environment for *Prevotella* growth^64^, but the pH in our study was maintained at a set point and so does not explain the outgrowth of this genus in the SPIME. It has been also reported that the donor’s endocrine system may impact the growth and activity of bacteria^30^including *Prevotella*;^65^ and that bile salts conversion can also influence the growth of some species of *Prevotella*^66–68^. In addition, the implementation of a standardized gut transit time (for technical reasons) in SPIME, which was slightly higher than the respective in vivo transit times may contribute to the proportional loss of aforementioned *Lactobacillus* or *Streptococcus* genera, while we previously showed longer transit times to select for higher *Prevotella* abundance^69^. Moreover, also the immune system and association with circulation system were excluded in SPIME’s design. In vivo, the primary bile acids are released in the proximal small intestine in response to fat content in the diet but are also affected by individual health conditions, as a physiological process^62,70^. In vitro, the bile salt concentration was selected based on what is considered physiologically relevant. The bile salts used for the in vitro experiments were commercially obtained and were processed products of adult porcine bile; its composition likely differs slightly from the bile of the four piglets used in this study. This will affect the levels of bile salts and their subsequent conversion; consequently, it may affect the growth of *Prevotella*, although further research is required. The absence of a host environment, together with the digestive and dietary parameters set in SPIME, collectively contributed to a strong selection of microbial species that were derived from the fecal samples and eventually grew in the respective SPIME gut compartments.

Despite the lower species richness in SPIME than in the in vivo community, 96% of named metabolites identified from the in vivo were also measured in at least one sample from the SPIME communities, so metabolite differences were quantitative rather than absences (Fig. 5, Supplementary Table 4). The trait-based genome-resolved functional profiling (Supplementary Fig. 4) and the CAZyme determination (Supplementary Fig. 5) also showed that the loss of taxa only exerts relatively small effects on the overall functional potential of SPIME community. This is consistent with the results of alpha-functional diversity analysis, by which the overall functional potential as measured by KEGG orthologs (KOs) in SPIME was similar to or higher than that in vivo communities (Fig. 2C), despite the SPIME possessing fewer taxa. This suggested functional redundancy, and potentially the taxa favored by the in vitro conditions possessed more diverse metabolic repertoires (more KOs), as shown by the Shannon Index of KO (Fig. 2D), and thus compensated for the loss of other taxa.

Only 64 microbial species persisted above detection limits from the in vivo community to the SPIME via the fecal samples (Supplementary Tables 3 and 5, Supplementary Fig. 6), which represents the functional continuity found across the experiments since they confer the same functional potentials to the SPIME community as detected for the whole in vivo community. While these functions are present in the persistent species, they are most likely further redundantly encoded by other members of the in vitro communities. Between-community functional redundancy requires shared taxa between the communities. Essentially, this means that different microbial communities can perform same functions, even if a part of their composition are taxonomically distinct. These shared taxa allow for functional redundancy across different communities and are crucial to ecosystem stability and resilience^71^. Thus, the 64 species, selected by environmental perturbation are considered the determining bacterial set of both the in vivo and the SPIME communities. That implies that the microbial community of the SPIME might be able to support a wider range of metabolic functions, potentially making it more adaptable and resilient to environmental changes.

While functional redundancy may protect microbial communities from functional losses resulting from species losses, it doesn’t eliminate quantitative differences (Fig. 4A). There are many elements that can affect the quality of metabolites. Our analysis allows us to look at variations in taxa and gene abundances, but a single gene can code for an enzyme with multiple isozymes. These isozymes can act on the same substrate to produce the same product, but may exhibit slightly different activities, efficiencies, or regulatory properties. Tryptophan can be catalyzed to N-formylkynurenine by two isozymes, tryptophan 2,3-dioxygenase and indoleamine 2,3-dioxygenase. The catalytic efficiency differs from each other, affecting the conversion rate and the overall tryptophan metabolism and the production of downstream metabolites^72–74^. The atoD gene (K01034), examined in Fig. 4E, is another example. It encodes the alpha subunit of acetate CoA/acetoacetate CoA-transferase. This enzyme has two isozymes, butyrate-acetoacetate CoA-transferase and butanoyl-CoA: acetoacetate CoA-transferase, both catalyze the transfer of CoA from butanoyl-CoA to acetoacetate, resulting in the production of butanoate (butyrate) and acetoacetyl-CoA with slightly different activity^75^. Genetic variation among microbial strains can lead to differences in enzyme activity and metabolite production, even while the overall function remains the same^76,77^. The transition from the in vivo conditions to the SPIME alters the microbial community composition (Fig. 4 and Supplementary Fig. 1) via environmental changes (lack of immune system and endocrine response, shifts in physiological conditions and digestive secretions), and consequently, this varies the interaction between bacteria, all together creating a micro-environment that influence microbial activity. In addition to broad scale changes which may change metabolite flux through a system, the lack of metabolite uptake by the host system can contribute to observed proportional differences in metabolite yields. Some of these were partially described or discussed by previous publications^22–26^.

Functional redundancy is an important part of the stability of the gut ecosystem^78–80^and this model would likely overestimate the detrimental effect of taxonomic loss since a smaller number of taxa are responsible for the functional stability of the whole community compared to in vivo. However, it is still unclear if the microbial communities in the SPIME and the piglets are similar enough to accurately represent pig gut microbial dynamics in response to a wide array of perturbations. While this experiment has demonstrated the ability to functionally recapitulate the piglet gut microbiome in vitro, future research with specific dietary interventions or perturbations can further elucidate to what extent the simulated gut microbiota display the same responsiveness to dietary alterations, antibiotic disturbances or pathogen intrusions.

## Electronic supplementary material

Below is the link to the electronic supplementary material.

Supplementary Material 1.

Supplementary Material 2.

Supplementary Material 3.

Supplementary Material 4.

## Data Availability

Shotgun metagenomic sequencing data has been deposited in the NCBI Sequence Read Archive associated with BioProject PRJNA1163371.
