# Supplementary material for: Structural and functional characterization of a porcine intestinal microbial ecosystem developed in vitro
Source: Sci Rep. 2025 Jul 10;15:24821. doi: 10.1038/s41598-025-10144-5 (PMC12246222; doi:10.1038/s41598-025-10144-5)
Supplement: Supplementary file 1 — Supplementary Material 1. [file 41598_2025_10144_MOESM1_ESM.docx]

**Supplementary information for:**

**Structural and functional characterization of a porcine intestinal microbial ecosystem developed *in vitro***

LinShu Liu^1,*^, Jenni A. Firrman^1^, Adrienne B. Narrowe^1^, Karley K. Mahalak^1^, Johanna MS Lemons^1^, Massimo Marzorati^2^, Cindy Duysburgh^2^, Chloë Rotsaert^2^, Eline Declerck^2^, Mattia Van den Broeck^2^, Chelsea Vannieuwenhuyse^2^, Renko Verstraete^2^, Amélie Van De Weghe^2^, Pieterjan Desmet^2^, Tom Van de Wiele^3,4^

^1^Eastern Regional Research Center, Agricultural Research Service, United States Department of Agriculture, Wyndmoor, PA 19038, United States of America.

^2^ProDigest, 82 Technologiepark, 9052 Gent, Belgium

^3^ Center for Microbial Ecology and Technology, Ghent University, Frieda Saeysstraat 1, B-9052 Gent, Belgium

^4^ International Associated Labs – HoMiGut, Ghent University, Belgium and University of Clermont Auvergne, France

**Corresponding author :* [linshu.liu@usda.gov](mailto:linshu.liu@usda.gov)

**Supplementary Figure 1** Cladogram showing results of LefSe analysis for taxa differing significantly between *in vivo* and *in vitro* samples. Members of the *Lactobacillaceae* are the most changed; (S1B) Family level relative abundances.


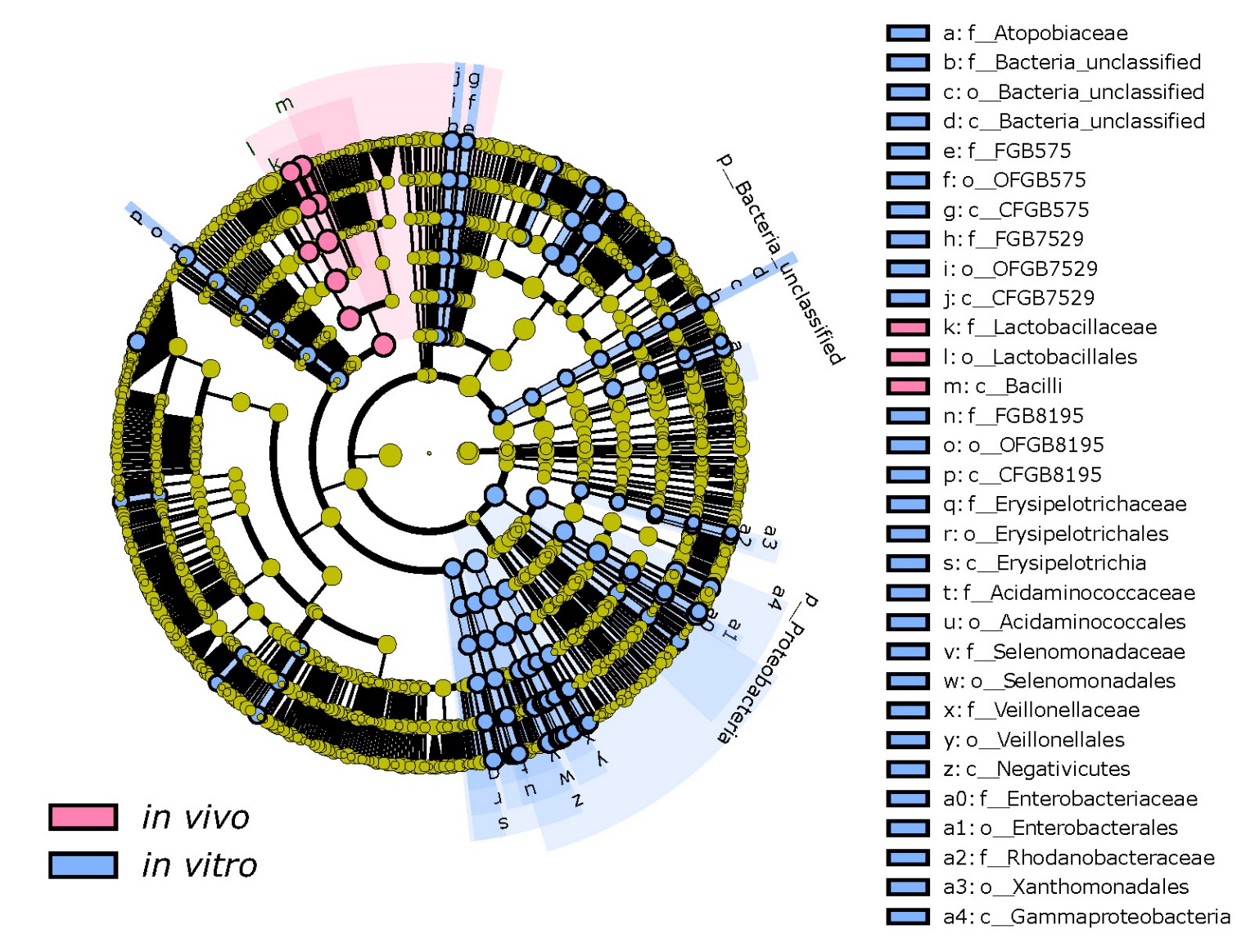


**Supplementary Figure 2** Family level relative abundances


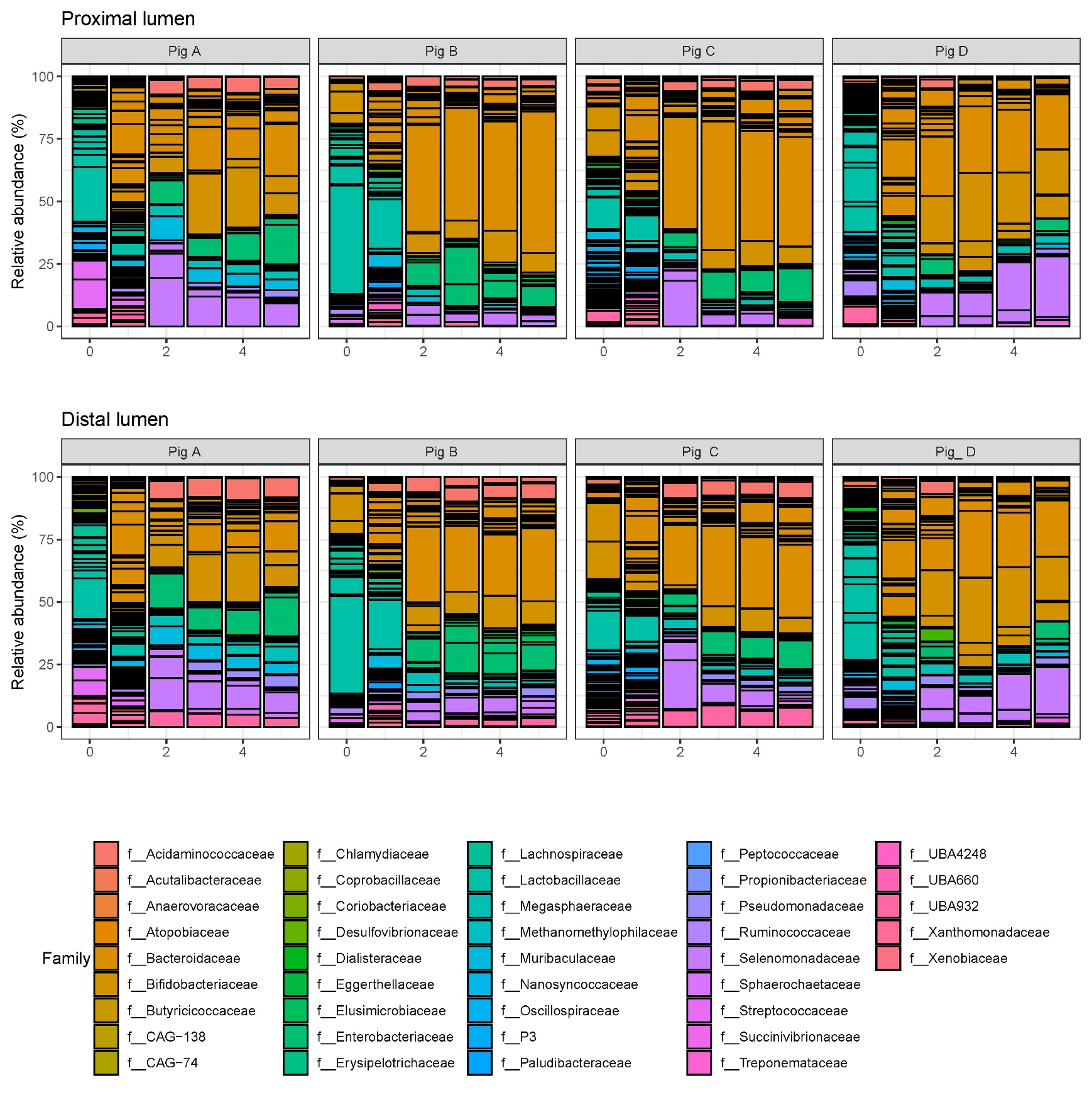


**Supplementary Figure 3** Relative abundances of two of the most changed families from *in vivo* to *in vitro.* Colors show the genera within each family type


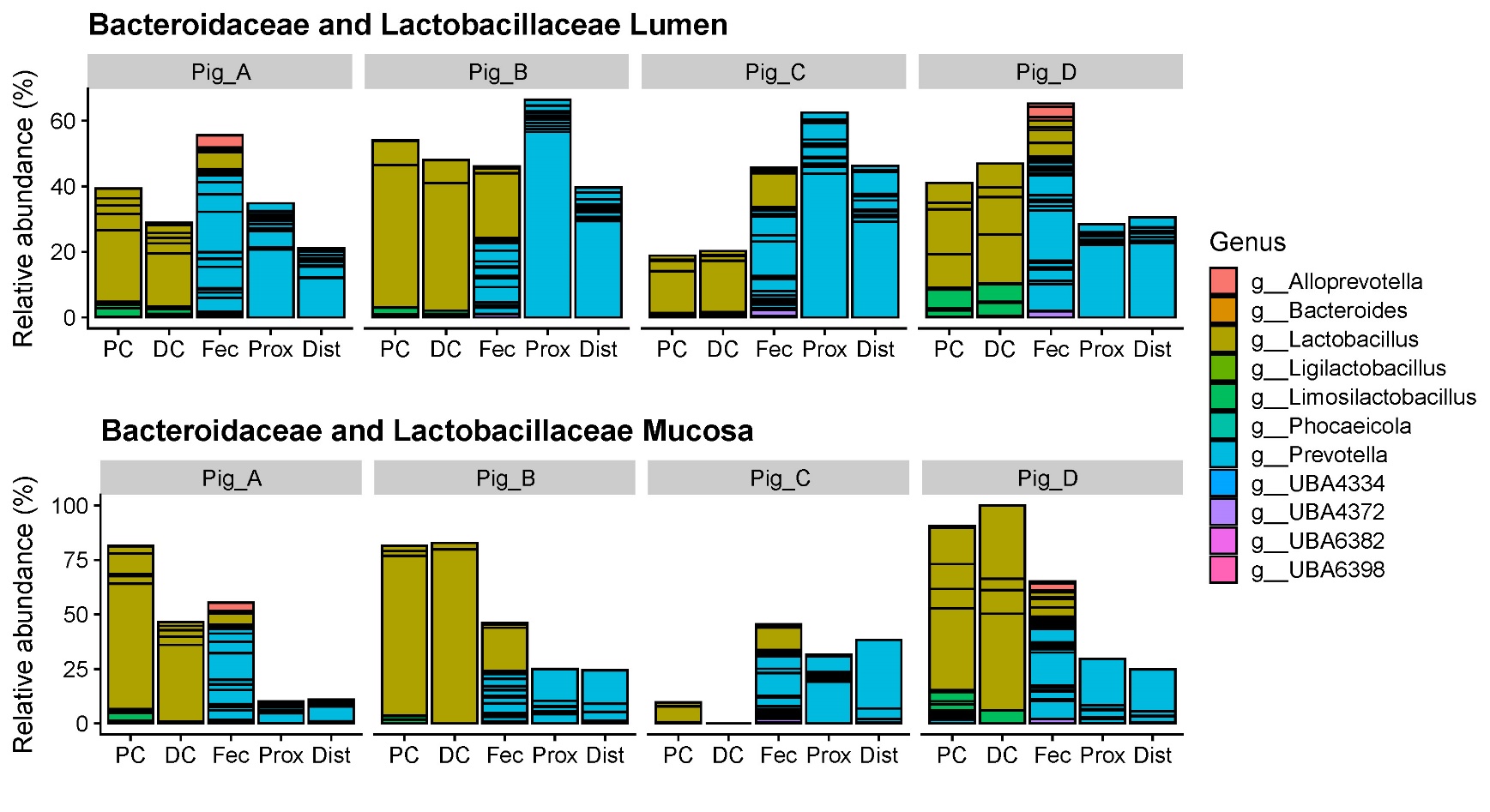


**Supplementary Figure 4** Heatmap showing trait abundances across the samples. Arrows indicate areas described in the text.
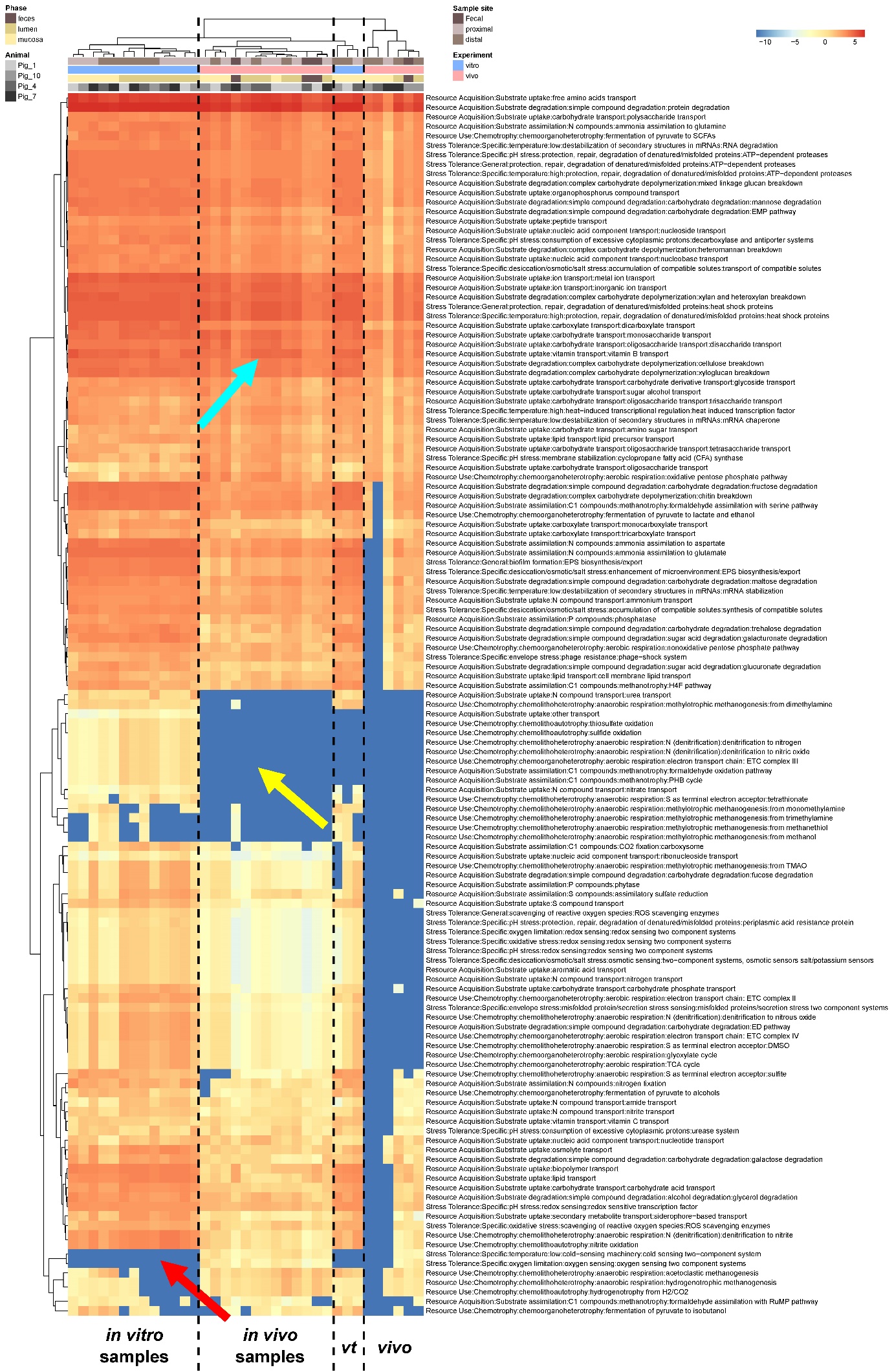


**Supplementary Figure 5** Heatmap showing distribution of CAZYme substrate utilization potential as determined from MAGs. Values are sums of per-sample MAG x substrate abundances. Columns represent individual samples, rows represent substrates.


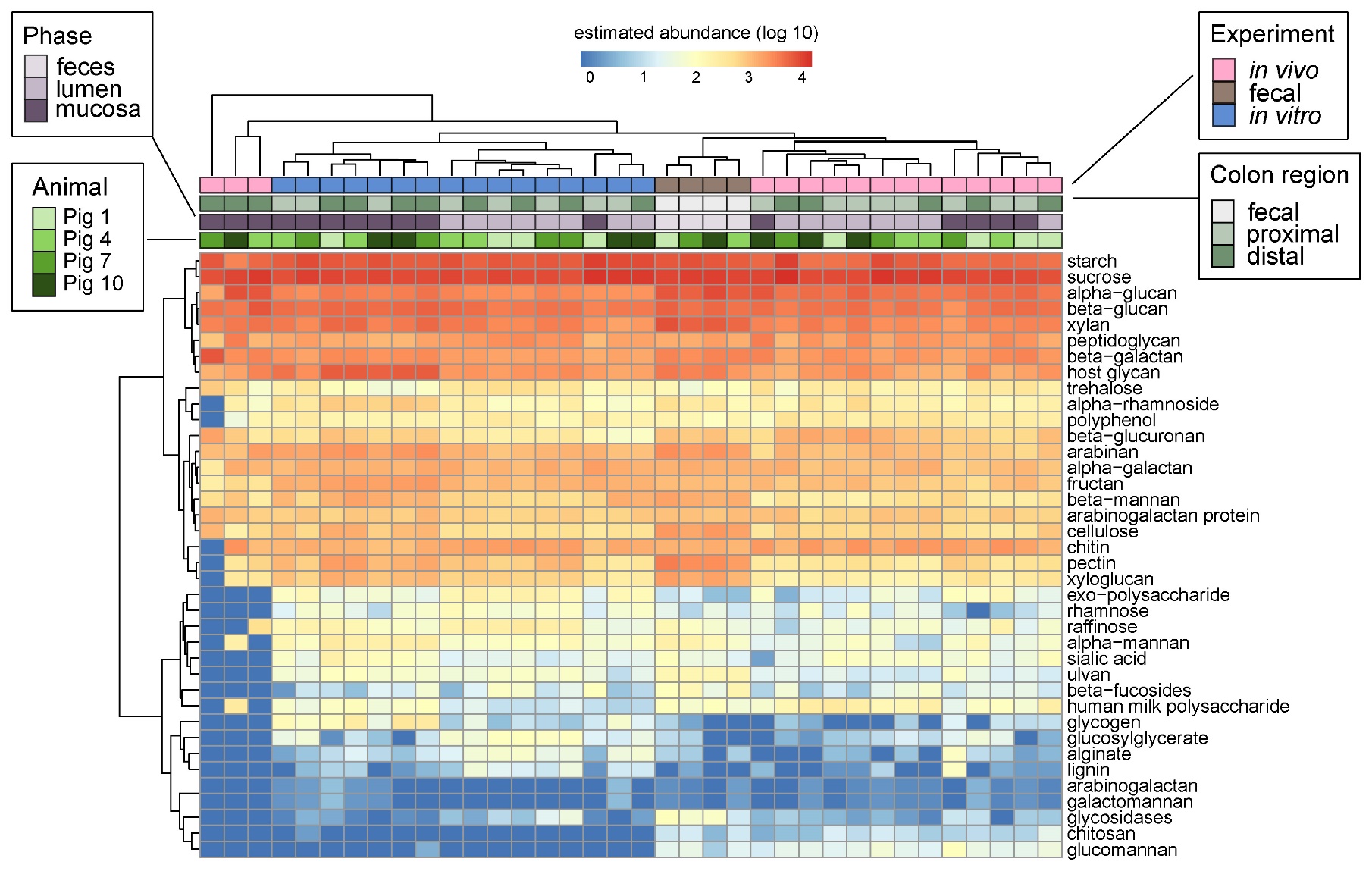


**Supplementary Figure 6.** Heatmap showing trait abundances of the genomic representatives of the 64 shared species in the *in vivo* and *in vitro* samples. **
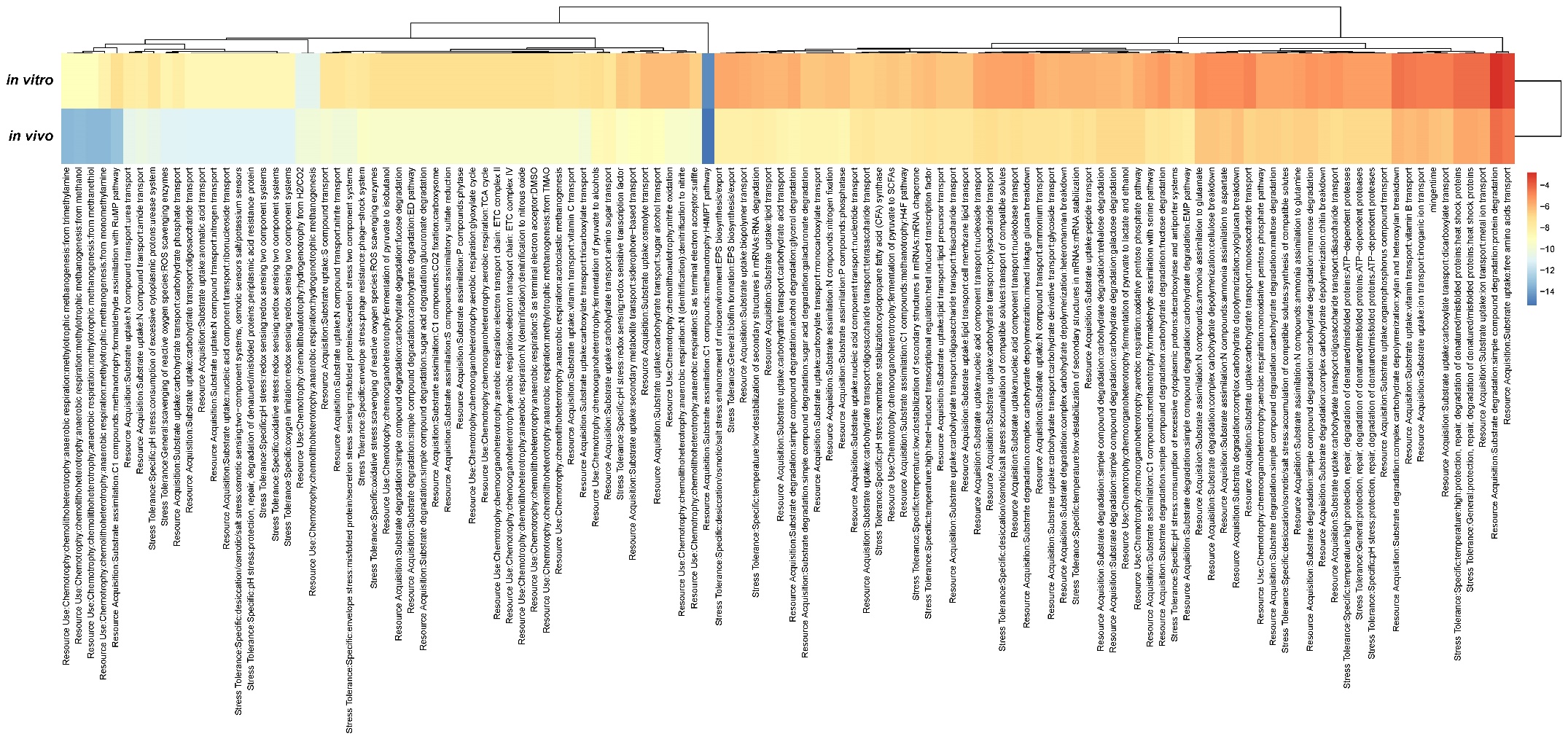
**

**Supplementary Table 1.** Composition of experimental diet (as-fed basis).

| Ingredient *g kg^-1^* |  |
| --- | --- |
| Corn starch | 337 |
| Wheat bran | 150 |
| Fish meal (68% crude protein) | 120 |
| Sweet Weij powder (75% lactose) | 100 |
| Potato protein (77% crude protein) | 66 |
| Oat hulls | 50 |
| Sugarbeet pulp | 50 |
| Dextrose | 50 |
| Arbocel (source of cellulose) | 20 |
| Animal fat | 20 |
| Soy oil | 20 |
| Vitamin and trace mineral premix ^1^ | 5.0 |
| Limestone | 4.7 |
| Sodium bicarbonate | 2.6 |
| DL-Methionine | 1.4 |
| Monocalcium phosphate | 1.3 |
| L-Tryptophane | 0.7 |
| L-Threonine | 0.7 |
| L-Lysine-HCl | 0.6 |
| Nutrient, *g/kg* |  |
| Dry matter | 904 |
| Starch | 330 |
| Sugar | 137 |
| Crude fiber | 48 |
| Non-starch polysaccharides | 157 |
| Crude protein | 180 |
| Ash | 50 |
| Crude fat | 61 |

^1^ Premix providing per kg of diet: vitamin A (retinyl acetate), 10000 IU; vitamin D3 (cholecalciferol), 2000 IU; vitamin E (dl-α-tocopherol acetate), 120 mg; vitamin K3 (menadione), 1.5 mg; vitamin B1 (thiamin), 1.0 mg; vitamin B2 (riboflavin), 4.0 mg; niacin, 30 mg; D-pantothenic acid, 15 mg; vitamin B6 (pyridoxin-HCl), 1.5 mg; vitamin B12 (cyanocobalamin), 20 µg; folic acid, 0.4 mg; biotin, 0.05 mg; choline chloride, 150 mg; Fe (FeSO_4_.H_2_O), 100 mg; Cu (CuSO_4_.5H_2_O), 120 mg; Mn (MnO), 30; Zn (ZnSO_4_.H_2_O), 70 mg; I (KI), 0.7 mg; Se (Na_2_SeO_3_), 0.25 mg

**Supplementary Table 2.** Excel file containing PERMANOVA statistics associated with Figure 3.

**Supplementary Table 3.** Shared species (as determined by MAGs and read-based analysis) for the fecal, in vivo, and in vitro samples as shown in Figure 4.

**Supplementary Table 4.** Excel file containing normalized metabolite data associated with Figure 5. Column A (Map order) indicates the order of the metabolites as shown from right to left in Figure 5. Values shown represent the average areas for each of the two experiments.

**Supplementary Table 5**. Per-sample relative abundances of 64 species shared across experiments.

| Phylum  (Bacteria / Archaea) | Number of species | Average per-sample relative abundance, % | | |
| --- | --- | --- | --- | --- |
|  |  | SPIME | Fecal inoculum | *In vivo* origin |
| **Actinomycetota (B)** | 11 | 11.35 ± 9.70 | 0.33 ± 0.91 | 6.15 ± 9.63 |
| **Bacteroidota (B)** | 19 | 7.26 ± 6.05 | 1.18 ± 2.20 | 5.12 ± 9.92 |
| **Bacillota (B)** | 30 | 16.33 ± 14.53 | 0.45 ± 0.80 | 3.22 ± 3.08 |
| **Proteobacteria (B)** | 1 | 4.68 ± 4.28 | 0.17 ± 0.09 | 0.41 ± 0.64 |
| **Desulfobacteriota (B)** | 1 | 0.64 ± 0.62 | 0.23 ± 0.22 | 0.26 ± 0.47 |
| **Methanobacteriota (A)** | 1 | 0.003 ± 0.019 | 0.002 ± 0.004 | 0.002 ± 0.005 |
| **Thermoplasmatota (A)** | 1 | 1.27 ± 3.15 | 0.07 ± 0.05 | 0.02 ± 0.04 |
